# Chromosome-level genome assembly of *Phrynocephalus forsythii* using third-generation DNA sequencing and Hi-C analysis

**DOI:** 10.1093/dnares/dsad003

**Published:** 2023-03-06

**Authors:** Yue Qi, Wei Zhao, Yangyang Zhao, Chenkai Niu, Shuhui Cao, Yingmei Zhang

**Affiliations:** Gansu Key Laboratory of Biomonitoring and Bioremediation for Environmental Pollution, School of Life Sciences, Lanzhou University, Lanzhou, China; Gansu Key Laboratory of Biomonitoring and Bioremediation for Environmental Pollution, School of Life Sciences, Lanzhou University, Lanzhou, China; Gansu Key Laboratory of Biomonitoring and Bioremediation for Environmental Pollution, School of Life Sciences, Lanzhou University, Lanzhou, China; Gansu Key Laboratory of Biomonitoring and Bioremediation for Environmental Pollution, School of Life Sciences, Lanzhou University, Lanzhou, China; Gansu Key Laboratory of Biomonitoring and Bioremediation for Environmental Pollution, School of Life Sciences, Lanzhou University, Lanzhou, China; Gansu Key Laboratory of Biomonitoring and Bioremediation for Environmental Pollution, School of Life Sciences, Lanzhou University, Lanzhou, China

**Keywords:** *phrynocephalus forsythii*, chromosome-level genome, karyotypes evolution, extreme environments adaptation

## Abstract

*Phrynocephalus forsythii* is a viviparous sand lizard that is endemic to the Tarim Basin with a broad altitudinal range of 872–3,100 m. Such variation in altitude and ecological variables can offer an opportunity to uncover genetic mechanisms of ectothermic adaptation to extreme environments at high- and low-altitude. Furthermore, the evolutionary relationship of karyotype with two different chromosome numbers (2*n* = 46 or 2*n* = 48) in the Chinese *Phrynocephalus* is unclear. In this study, a chromosome-level reference genome of *P. forsythii* was assembled. The genome assembly size was 1.82 Gb with a contig N50 length of 46.22 Mb, 20,194 protein-coding genes were predicted and 95.50% of these genes were annotated in functional public databases. After cluster contigs into chromosome level using Hi-C paired-end reads, we found that two chromosomes of *P. forsythii* were originated from one ancestral chromosome of species with 46 chromosomes. Comparative genomic analysis revealed that numerous characteristics associated with high- or low-altitude adaptation, including energy metabolism pathways, hypoxic adaptation, and immune, exhibit rapid changes or show signals of positive selection in the *P. forsythii* genome. This genome provides an excellent genome resource for the study of the karyotype evolution and ecological genomics of *Phrynocephalus.*

## 1. Introduction

*Phrynocephalus* is one of the major components of the central Asian desert fauna. Chinese *Phrynocephalus* species form two distinct clades: lowland oviparous and highland viviparous clade, and the karyotype between these two clades are different, which can be divided into two types according to the number. The first type is species with 46 chromosomes, all other lowland oviparous species belong to this category apart from *P.mystaceu*s. The karyotype characteristic of these species is 2*n* = 46, with 11 pairs of macrochromosomes and 12 pairs of microchromosomes. The second type is species with 48 chromosomes, all highland viviparous clade species and *P. mystaceus* belong to this category. The karyotype characteristic of these species is 2*n* = 48, among which viviparous clade species have 12 pairs of macrochromosomes and 12 pairs of microchromosomes, while *P. mystaceus* had 11 pairs of macrochromosome and 13 pairs of microchromosomes.^1^ However, the sequence of karyotype evolution among Chinese *Phrynocephalus* has not been resolved.

**Figure 1.**
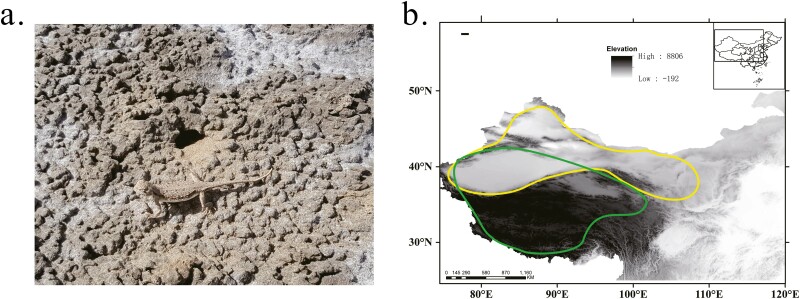
(A) photograph of *Phrynocephalus forsythii* from Xinjiang Uygur Autonomous Region. (B) The distribution of the Chinese *Phrynocephalus* species. The yellow circle represents the distribution regions of the lowland oviparous clade species and the green circle represents the distribution regions of the highland viviparous clade species.

Determining the adaptability of Chinese *Phrynocephalus* species to extreme environments is also of scientific interest. As ectothermic, *Phrynocephalus* species' body temperature is highly dependent on the environment, and therefore faces physiological challenges due to inhabiting habitats with extremely high- or low-temperature environments. Chinese *Phrynocephalus* is widely distributed, which spans from arid regions of northwestern China to the Qinghai-Tibet Plateau (Fig. 1). Therefore, for highland viviparous species that are primarily restricted to the Qinghai-Tibet Plateau, low ambient temperatures, hypoxic environment and increased ultraviolet light exposure are the major threat to their survival.^2–4^ For lowland oviparous species that are mainly distributed in low altitude arid regions of northwestern China, high ambient temperatures are seen as mainly survival stress.^5,6^ In fact, *Phrynocephalus* has been identified as at high risk of total extinction due to thermal limits being exceeded.^7^ However, how Chinese *Phrynocephalus* species adapt to these extreme environments at the genome level has not been reported.

*Phrynocephalus forsythii* (family: Agamidae), a sand lizard with a unique distribution and phylogenetic location in the Chinese *Phrynocephalus*, possesses research value for karyotype evolution and extreme environment adaptation research. *Phrynocephalus forsythii* is the first divergent species in highland viviparous clade,^8^ as a transitional species between lowland oviparous and highland viviparous clade of Chinese *Phrynocephalus*, *P. forsythii* genome may provide some valuable information related to the karyotypes evolution. Furthermore, *P. forsythii* exhibits a broad distribution with range of 872–3,100 m, this distribution pattern was due to the ancestor of *P. forsythii* isolated on the Qinghai-Tibet Plateau, and then spread to the Tarim Basin.^8,9^ Therefore, *P. forsythii* may highlight the ancestral high-altitude environment adaptation state of *Phrynocephalus*. Besides, since *P. forsythii* subsequently spread and colonized to the northern Tarim Basin, a arid and semi-arid region with high temperature and low precipitation, this species may also underwent an adaptation to high-temperature environment. Our previous study indicated that the genetic diversity of *P. forsythii* population distributed in the northern Tarim Basin was lower than that of high, hint that the adaptability of population was lower under high-temperature environment.^10,11^ We thus consider that molecular mechanisms related to adaptation of high ambient temperatures can also be explored in the genome of *P. forsythii.*

In this study, we combined genomic sequencing data from Illumina short reads, Oxford Nanopore long reads, and Hi-C data to generate a chromosome-level reference genome of *P. forsythii*, which has 48 chromosomes.^1^ We also downloaded the genome of *P. perzewalskii*,^12^ which belongs to Chinese *Phrynocephalus* lowland oviparous clade and has 46 chromosomes.^1^ We compared the two genomes to explore the karyotype relationship between the Chinese *Phrynocephalus* oviparous clade species with 46 chromosomes and viviparous clade species. We further annotated the protein-coding genes in the genome and used the comparative genome approach to verify how *P. forsythii* adapt to the extreme environment about high- and low-temperature environment. This genome provides an excellent resource for karyotype evolution and extreme environment adaptive evolution research of Chinese *Phrynocephalus.*

## 2. Materials and Methods

### 2.1. Ethics statement

All experiments were approved by the Ethics Committee of the School of Life Sciences, Lanzhou University and followed the committee guidelines.

### 2.2. Sample collection and sequencing

Adult female *P. forsythii* was collected by hand from Korla (Xinjiang Uygur Autonomous Region of China), at an elevation of 876 m, in the north of Tarim Basin in July 2019. Fresh tissues, including the heart, liver, brain, gonad, and kidney, were separated and immediately immersed in liquid nitrogen. All tissues were subsequently stored in a drikold and returned to the lab. Liver tissue was used to extract DNA for genome sequencing, and all of these inner visceral organs were used for RNA-sequencing (RNA-seq) analysis and genome annotation.

Short-read sequencing, long-read sequencing, and Hi-C sequencing technologies were used to obtain the *P. forsythii* genome information. For short-read sequencing, a short insert library with a size of 350 bp was constructed using the TruSeq Library Construction Kit and was then sequenced on an Illumina NovaSeq 6000 Sequencing System (Illumina Inc., San Diego, CA, USA). Raw reads with >10% unknown bases or with >20% low-quality bases and all adaptor sequences produced in the PCR process will be removed as low-quality sequences. For long-read sequencing, another library was constructed using the genomic sequencing kit SQK-LSK108 and sequenced using the Promethion platform (Oxford Nanopore Technologies, Oxford, UK). Generated nanopore raw reads <1 kb in length or with a mean quality value of <7 were removed. The Hi-C library was constructed for the chromosome assembly of *P. forsythii.* Cells form liver tissue were cross-linked using the formaldehyde solution, and the DNA was cut using the HindIII enzyme. Biotinylated nucleotides were used to mark ends of restriction fragments and the T4 Ligase was used to ligate. Subsequently, the ligated DNA was sheared into a length of 350 bp and sequenced through the Illumina NovaSeq 6000 Sequencing System (Illumina Inc., San Diego, CA, USA) and the low-quality Hi-C (high-throughput chromosome conformation capture) reads were filtered using HiC-Pro v2.10.0 with default parameters.

RNA libraries were constructed to assist in genome annotation. RNA sequencing was performed on five tissues (heart, liver, brain, ovaries, and kidney) obtained during sampling. The RNA-seq library was constructed using the NEBnext Ultra-Directional RNA Library Prep kit (NEB, protocol B) and sequenced on the Illumina HiSeq 2000 platform.

### 2.3. Genome characteristic estimation and assembly

Illumina short reads were first used to estimate genome size. The 17-mer depth frequency distribution and total *k-mer* number were calculated using Jellyfish (v2.2.6)^13^ and SOAPdenovo.^14^ Genome size was estimated using the equation: genome size = *K_num/K_depth*, where *K_num* is the total *k-mer* number and *K_depth* is the peak *k-mer* frequency depth of 17-mer.

Nanopore long reads were employed to assemble primary genomes using wtdbg2 using the following parameters: -k 0 -p 21 -K 1000.049988 -A -S 4.000000 -s 0.050000 -g 0 -X 50.000000 -e 3 -L 0.^15^ To further improve the quality and accuracy of our genome assembly, the assembled genomic sequences were first polished by Racon v1.2.1 using Nanopore long reads and then polished by Pilon v1.21^16^ using Illumina short reads with the parameters of -Xmx30g—diploid—changes. After the genome was initially assembled and polished, Hi-C data were used to complete the assisted assembly of a chromosome-level genome. We used the ALLHiC scaffolding method to identify chromosomal Hi-C interactions, linking contigs using the following parameters: enz = MboI shortest_ = 150 MaxLinkDensity = 3 minREs = 50 break = Y filter = yes CLUSTER = 24 NonInformativeRatio = 0.^17^

### 2.4. Genome assembly evaluation

Three strategies were used to evaluate the quality and accuracy of the assembled genome. The integrity of the genome was first evaluated using BUSCO (v3.0.2b).^18^ Subsequently, the number of conserved eukaryotes present in the assembled genome of *P. forsythii* was determined using the Core Eukaryotic Genes Mapping Approach (CEGMA).^19^ Finally, all filtered Illumina short reads were aligned to the assembled genome using BWA to evaluate genome completeness.^20^

### 2.5. Genome annotation

Repeat sequences (tandem repeats and transposable elements [TEs]) within the genome were annotated according to different strategies. Tandem repeats were identified using Tandem Repeat Finder. TEs were identified through a combined strategy of the homolog and *de novo* prediction at both the DNA and protein levels. At the DNA level, homolog prediction was first implemented using the Repbase database to extract repeat regions. The *de novo* repetitive elements database was built using long tandem repeats (LTR)_FINDER, RepeatScout, and RepeatModeler. All repeat sequences possessing lengths >100 bp and gap Nʹ values <5% constituted the raw TE library. After prediction, a combination of the homolog alignment library and the *de novo* prediction library was supplied to RepeatMasker for repeat identification.^21^ At the protein level, RepeatProteinMask was used to search against the TE protein database.

Protein-coding genes within the entire genome were annotated using *de novo* prediction, homology-based prediction, and RNA-Seq-assisted prediction. For *de novo* prediction, Augustus v3.2.3, Geneid v1.4, GENESCAN v1.0, GlimmerHMM v3.04,^22^ and SNAP^23^ were used in our automated gene prediction pipeline. For the homology-based prediction, the protein sequences of *Salvator merianae*, *Anolis carolinensis*, *Lacerta viridis*, *Lacerta bilineata*, *Shinisaurus crocodilurus*, *Pogona vitticeps*, *Phrynocephalus przewalskii*, and *Phrynocephalus vlangalii* were downloaded from Ensembl and NCBI. The protein sequences of the above species were aligned to the genome of *P. forsythii* using TblastN v2.2.26 (*E*-value < 1e−5),^24^ and the matching proteins were then analysed for accurate spliced alignments using GeneWise v2.4.1.^25^ For RNA-Seq-assisted prediction, transcriptome read assemblies were first generated using Trinity v2.1.1.^26^ RNA-seq-based gene prediction was performed using PASA v2.0.2. Finally, a non-redundant reference gene set was generated by merging the genes predicted by the above three methods using EvidenceModeler v1.1.1.^27^

Gene functions were first assigned using InterProScan v5.31^28^ by searching against six different public protein databases, including Pfam v27.0,^29^ PRINTS v42.0, PROSITE v20.97, ProDom v2006.1, SMART v6.2, and PANTHER v12.0. Gene Ontology (GO) IDs for each gene were assigned according to the corresponding InterPro entries. Additionally, the SwissProt and NR databases were also used for functional annotation, and the gene sets were mapped to a KEGG pathway to identify the best match for each gene.

The tRNAs were predicted using tRNAscan-SE v2.0.^30^ For rRNAs, we chose relative species rRNA sequences as references and predicted rRNA sequences using BLAST. Small nuclear RNA (snRNA) and microRNA were annotated using the non-coding database Rfam v14.0.^31^

### 2.6. Genome alignment and synteny analysis

In order to explore the karyotypes relationships between Chinese *Phrynocephalus* species, MUMmer’s NUCmer alignment software was applied to compare the genome of *P. forsythii* (2*n* = 48) and *P. przewalskii* (2*n* = 46).^32^*P. przewalskii* scaffold-level genome (scaffold N50 = 6.88 Mbp) was downloaded from BIGD Genome Warehouse, bigd.big.ac.cn/gwh (accession nos. GWHAAFD00000000).^12^ Genome comparisons were carried out to produce delta files with default parameters.

Given that *P. przewalskii* genome is fragmented and synteny is generally well conserved beyond genera, we downloaded a chromosome-level genome of *Phrynosoma platyrhinos* form NCBI (No. PRJNA685451), which has six macrochromosomes and 11 microchromosomes.^33^ Furthermore, *P.platyrhinos* shared the most recent common ancestor with *P. forsythii* about 150 Mya (timetree.org), this species is an evolutionarily closer reptile to *P. forsythii* with a chromosome-scale assembly genome. We performed synteny analysis between *P. forsythii* and *P.platyrhinos* using MCscanx.

### 2.7. Comparative genome analysis

#### 2.7.1. Species-specific genes

In order to explore the adaptation characteristics of *P. forsythii* in both high- and low-altitude environments at the genome level, species involved in the analysis were selected according to different research purposes. For *P. forsythii* high-altitude adaptation characteristic analysis, *P. forsythii* and 8 other low-altitude species *Pseudonaja textilis*, *Podarcis muralis*, *Zootoca vivipara*, *Anolis carolinensis*, *Pogona vitticeps*, *Phrynocephalus przewalskii*, *Xenopus tropicalis,* and *Leptobrachium leishanense* were selected and grouped them as the “high-altitude adaptation group”, all species in this group have a lower elevation distribution than *P. forsythii* (Supplementary Table S1); for *P. forsythii* low-altitude adaptation characteristic analysis, we selected *P. forsythii* and seven other high-altitude species *Rhinopithecus bieti*, *Bos grunniens*, *Pseudopodoces humilis*, *Phrynocephalus vlangalii*, *Nanorana parkeri*, *Bufo gargarizans,* and *Triplophysa tibetana* into a group and named it as the “low-altitude adaptation group”, these species have a higher elevation distribution than *P. forsythii* (Supplementary Table S1). Altitudinal range of these species were obtained from https://www.gbif.org/search.

Homology relationships (including orthologs and paralogs) among species were determined using OrthoMCL.^34^ Protein sequences of above species were acquired from Ensembl or NCBI and were filtered to retain the longest transcript. Sequences of fewer than 50 amino acids in length were removed. These retained sequences were aligned reciprocally with the BLASTP plug-in on NCBI with a threshold of *e*-value <1e−5. As *P. forsythii* may exhibit several unique characteristics for high- or low-altitude adaption, unique paralogs, and unclustered genes were identified as species-specific genes. Furthermore, in order to eliminate the disturbance of genetic differences caused by interspecific differences, species-specific genes of *P. forsythii* common to the high- and low-altitude groups were removed. GO terms that were significantly over-represented among *P. forsythii-*specific genes were identified using BiNGO^35^ in Cytoscape.^36^

#### 2.7.2. Expanded and contracted gene families

Single-copy gene families were retrieved from the OrthoMCL results as described above and used for phylogenetic tree construction. The protein sequences from each family were aligned using MAFFT v7.475.^37^ Aligned sequences were eliminated in poorly aligned positions and divergent regions using Gblocks v0.91.^38^ The best-fit amino acid substitution model was selected using ProtTest v3.4.2 under the corrected Akaike information criterion and Bayesian information criterion, and the JTT + I + G + F model was finally selected.^39^ Subsequently, maximum likelihood phylogenetic tree was constructed using RAxML v8.2.10.^40^ The MCMCTREE program in the PAML v4.8 was used to calculate divergence time.^41^ The fossil calibration records were acquired from the TIMETREE website. The MCMC process was executed for 6,000,000 iterations after a burn-in of 2,000,000 iterations. Expansion and contraction gene families were determined by CAFE v3.1. Similarly, genes common to the high- and low-altitude adaptation groups from *P. forsythii* expanded and contracted families were removed.

#### 2.7.3. Positively selected genes

To identify potential positively selected genes (PSGs) of *P. forsythii*, the gene family relationships between *P. forsythii* and three other species *(Anolts carolinensis, Phrynocephalus przewalskii,* and *Phrynocephalus vlangalii)* were calculated using the OrthoMCL. Protein sequences of each single-copy gene families were aligned using MAFFT v7.475 with default parameters. Subsequently, CDS alignments corresponding to those protein sequences were back-translated using PAL2NAL v14,^42^ and the conservative region of these CDS alignments were extracted using Gblocks v0.91. The branch-site model of CODEML in PAML v4.8 was selected to test the potential PSGs as *P. forsythii* was set as the foreground branch and the other species as background branches.

## 3. Results

### 3.1. Genome sequencing and assembly

**Figure 2.**
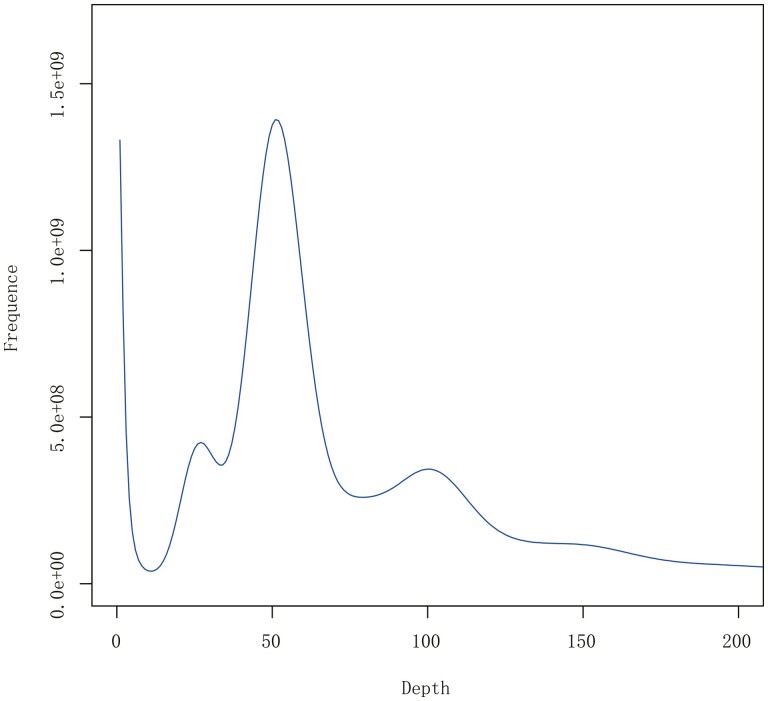
17-mer analysis of *Phrynocephalus forsythii* genome characteristics. The peak *k-mer* frequency depth of 17-mer was 50.

Genome of *P. forsythii* was sequenced through the Illumina platform, Oxford Nanopore platforms, and Hi-C technology and a total of ~206.92 Gb,~201.90 Gb, and ~208.26 Gb raw data were obtained, respectively (Table 1). According to the survey analysis, the total *k-mer* number was 94,714,458,115, and the peak *k-mer* frequency depth of 17-mer was 50 (Fig. 2). Finally, the estimated genome size was 1.89G.

**Table 1.** Sequencing data used for moustache toad genome assembly and annotation

Sequencing type	platform	Insert size (bp)	Raw data (Gb)	Clean data (Gb)	Sequence coverage (×)
Genome short reads	Iliumina Novaseq 6000	350	206.92	206.72	110.65
Genome long reads	PromethION	—	201.90	201.68	107.97
Genome Hi-C reads	Iliumina Novaseq 6000	—	208.26	207.91	111.37

The initial assembly of the genome was completed using the Nanopore long reads with N50 length of 19.58 Kb. After correcting the errors of the primarily assembled genome, a size of 1.82 Gb *P. forsythii* genome was assembled with the contig N50 was 46.22 Mb (Table 2), this genome size was basically consistent with the estimated genome size (1.89G).

**Table 2.** Assembly data for the *Phrynocephalus forsythii* genome

Term	Wtdbg contig		Hi-C scaffold	
	Size (bp)	Number	Size (bp)	Number
N90	7,786,151	54	26,992,927	15
N80	14,096,206	37	68,431,482	11
N70	21,821,924	26	111,457,702	9
N60	28,707,233	19	127,215,305	7
N50	46,215,561	14	133,255,571	6
Maximum length	95,027,530	—	212,878,235	—
Total length	1,816,870,809	916	1,816,900,611	645
No. ≥2,000 bp	—	942	—	642

**Figure 3.**
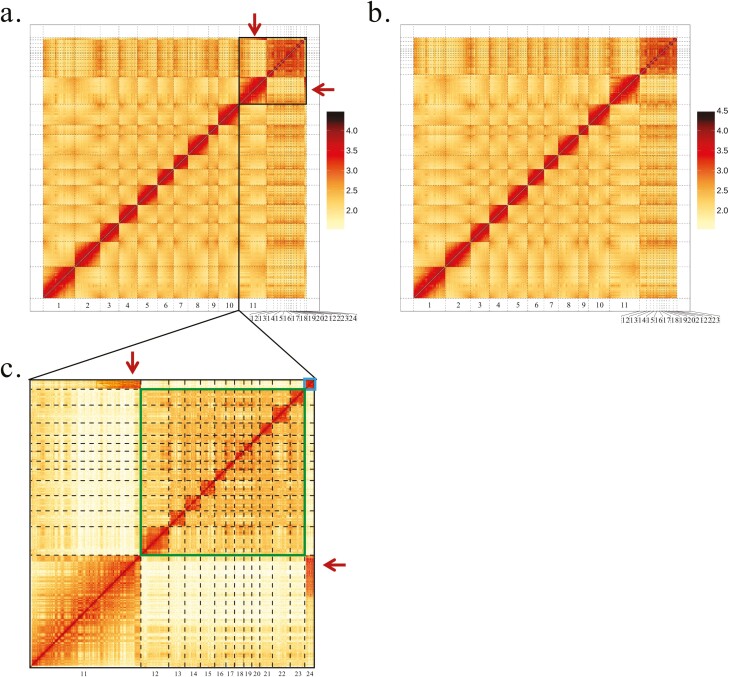
HiC-based heatmaps of DNA interaction of each chromosome. (A) Set K = 24, the red arrow indicates that two chromosomes are strongly interacting. (B) Set K = 23. (C) A close-up view of the Hi-C data marked with a black box in (A).

Previous karyotype analysis showed that *P. forsythii* had 48 chromosomes (2*n* = 48),^1^ we thus set *k* = 24 in the ALLHiC program to assemble the chromosome-level genome. Finally, ~207.91 Gb Hi-C clean data were mapped to the assembled contigs and 645 assembled contigs were mapped to 24 chromosomes, accounting for 99.35% of the entire genome, with the N50 lengths were 133.26 Mb (Fig. 3A, Table 2). However, Hi-C-based heatmaps of DNA interaction of each chromosome indicated that two chromosomes (chromosome 11 and chromosome 24) have stronger interaction (marked with a red arrow in Fig. 3C), we, therefore, re-assembled chromosomal-scale genome and observed that contigs were well phased into 23 homologous chromosomes when K was set to 23, with 99.36% of contigs were clustered into the 23 groups (Fig. 3B). Given that previous studies have reported that *P. forsythii* has 48 chromosomes, we thus used the genome with 24 chromosome-scale scaffolds assembled by Hi-C data as the final assembly and for the subsequent analysis.

### 3.2. Genome assembly evaluation

BUSCO assessment indicated the presence of 94.7% of complete BUSCOs, with 94.2% complete and single-copy BUSCOs, 0.5% complete and duplicated BUSCOs, 2.8% fragmented BUSCOs, and 2.5% missing BUSCOs. Using the CEGMA method, a total of 239 conserved core genes (96.37%) of the 248 core eukaryotic genes were identified within the genome. Furthermore, a total of 97.78% of clean short reads could be mapped to the genome, and these clean reads covered 98.67% of the genome. These results indicated that the assembled genome covered most of the genetic regions, further confirming the assembly quality of the *P. forsythii* genome.

### 3.3. Genome annotation

**Figure 4.**
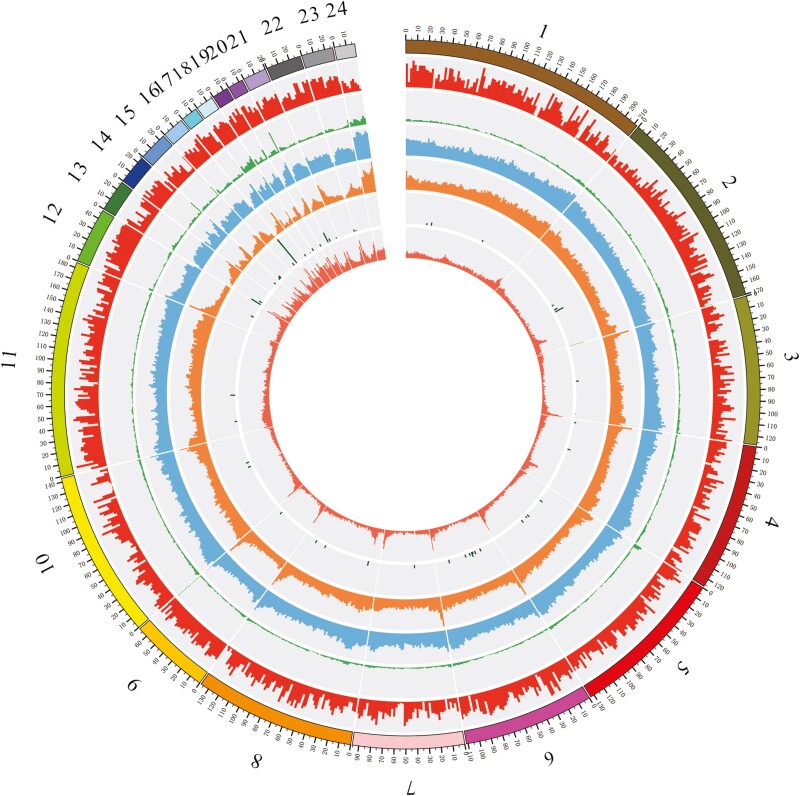
Circos graph showing characteristics of *Phrynocephalus forsythii* genome. From outer circle to inner ring: gene distribution, tandem repeats (TR), long tandem repeats (LTR), long interspersed nuclear elements (LINE), short interspersed nuclear elements (SINE), and guanine-cytosine (GC) content.

A total of 845.99 Mb of repeat sequences were obtained, and these accounted for 46.56% of the *P. forsythii* genome. Among the repeated sequences, 3.26% (59.16 Mb) were tandem repeats and 46.1% (837.68 Mb) were TEs. In the TEs, the long interspersed nuclear elements, short interspersed nuclear elements, DNA elements (SINEs) and long-terminal repeats account for 11.79%, 0.01%, 0.36%, and 40.97%, respectively (Table 3, Fig. 4).

**Table 3.** Statistics on TEs in *Phrynocephalus forsythii* genome

	*De novo*+Repbase		TE Proteins		Combined TEs	
	Length(bp)	% in Genome	Length(bp)	% in Genome	Length(bp)	% in Genome
DNA	5,866,319	0.32	968,798	0.05	6,563,784	0.36
LINE	59,506,416	3.28	195,548,238	10.76	214,132,108	11.79
SINE	235,439	0.01	0	0	235,439	0.01
LTR	743,367,281	40.91	16,810,953	0.93	744,309,116	40.97
Unknown	31,734,738	1.75	0	0	31,734,738	1.75
Total	826,087,623	45.47	213,313,385	11.74	837,681,520	46.1

**Figure 5.**
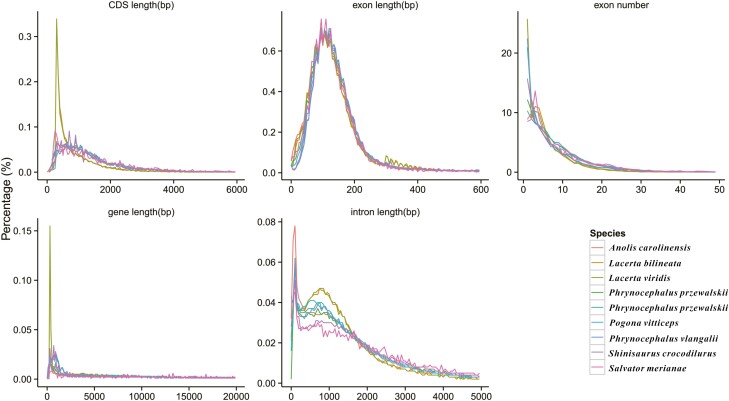
Annotation quality comparison of protein-coding genes. We compared the CDS length, exon length, exon number, gene length and intron length among 9 species.

A total of 20,194 protein-coding genes were obtained by combining the results of three different approaches. Furthermore, the distributions of CDS length, exon length, exon number, gene length, and intron length of the *P. forsythii* genome were comparable to those of the closely related species, with the exception of *Lacerta viridis* and *Lacerta bilineata*, which have a large number of short-length CDSs and genes. We think that this feature may be unique to *Lacerta* species and therefore consider *P. forsythii* genome annotation results were accurate (Fig. 5). Additionally, of these protein-coding genes, 19,290 genes were annotated from the functional databases and accounted for 95.50% of predicted protein-coding genes (20,194) of *P. forsythii* (Table 4).

**Table 4.** Functional annotation of predicted protein-coding genes

Term	Number	Percent (%)
Swissprot	17,877	88.5
Nr	19,197	95.1
KEGG	17,197	85.2
InterPro	18,091	89.6
GO	12,830	63.5
Pfam	16,071	79.6
Annotated	19,290	95.5
Unannotated	904	4.5
Total	20,194	100

For non-coding RNA, a total of 7,898 microRNAs, 33,614 rRNAs, 1,757 snRNAs, and 359 tRNA genes were annotated (Supplementary Table S2).

### 3.4. Genome alignment and synteny analysis

**Figure 6.**
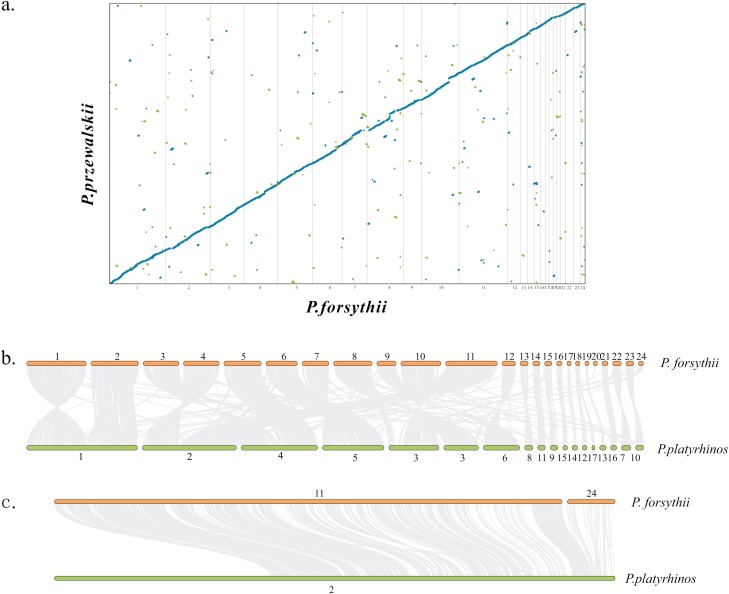
Genome alignment and synteny analysis. (A) Large-scale genome alignment of *P. forsythii* (2n=48) and *P.przewalskii* (2n=46). The x-axis denotes the 24 chromosomes of *P. forsythii*. The y-axis represents genomic scaffolds of *P.przewalskii*. Blue dots denote unique forward alignments. Green dots denote unique reverse alignments. (B) and (C) Synteny between genomic regions of *P. forsythii* and *P.platyrhinos*. The thin lines represent all collinear regions in the corresponding chromosomes. Bold font denotes the corresponding chromosomes in *P. forsythii* and *P.platyrhinos*, respectively. Among them, chromosome 3 of the *P.platyrhinos* did not assemble into one; (B Synteny between all chromosomes of *P. forsythii* and *P. latyrhinos*; (C) Synteny between *P. forsythii* chromosome 11 and 24 and *P. latyrhinos* chromosome 2.

We mapped scaffolds-scale genome of *P. przewalskii* to the chromosomes of *P. forsythii* to identify the karyotype relationship in the Chinese *Phrynocephalus* species. In total, 1.16 Gbp sequences (67%) of *P. przewalskii* genome were aligned to the genome of *P. forsythii.* The alignments reveal that although the two species have different numbers of chromosomes, most of *P. przewalskii* scaffolds are aligned with 24 chromosomes of *P. forsythii*, and exhibit a unique forward alignment (Fig. 6A).

Genome synteny analysis showed that most of microchromosomes of *P. forsythii* exhibit genome synteny with those of *P. platyrhinos.* However, *P. forsythii* chromosomes 24, another microchromosomes, together with chromosome 11 exhibited genome synteny with one macrochromosome of *P.platyrhinos* (Fig. 6B and 6C).

### 3.5. Comparative genome analysis

#### 3.5.1 Species-specific genes

**Figure 7.**
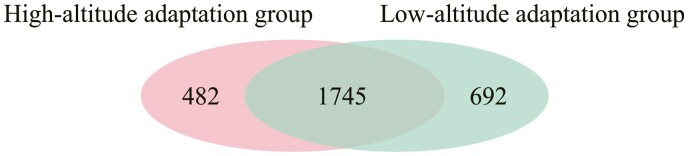
The count of *P. forsythii* species-specific genes obtained from high-altitude adaptation group and low-altitude adaptation group respectively.

To study the adaptive characteristics of *P. forsythii* in high altitude environments, we explored species-specific genes of *P. forsythii*. A total of 19,276 gene families and 10,986 unclustered genes were clustered in the nine species (Supplementary Table S3). Furthermore, 2,227 species-specific genes were identified. After removing species-specific genes common with the low-altitude group, 482 species-specific genes were identified (Fig. 7), these genes were over-represented in some major categories, such as DNA integration (corrected *P* = 2.48e−04), DNA metabolic process (corrected *P* = 1.67e−02), calcium-ion regulated exocytosis (corrected *P* = 2.48e−04), calcium-ion transport (corrected *P* = 5.68e−03) and calcium channel complex (corrected *P* = 5.34e−03) (Supplementary Table S4).

For the adaptation characteristics of *P. forsythii* to low-altitude environments research, a total of 20,643 gene families and 13,047 unclustered genes were clustered in the 8 species (Supplementary Table S3). Of them, 2,437 species-specific genes were identified and 692 were retained after filtered genes common with the high-altitude group (Fig. 7). These species-specific genes unique to low-altitude environments were over-represented in some major categories, such as immune response-activating cell surface receptor signalling pathway (corrected *P* = 1.25e−03), B-cell receptor signalling pathway (corrected *P* = 1.25e−03), immune response-activating signal transduction (corrected *P* = 1.25e−03), ATPase inhibitor activity (corrected *P* = 4.47e−02), negative regulation of ATP-dependent activity (corrected *P* = 4.50e−02) and regulation of ATP-dependent activity (corrected *P* = 4.50e−02) (Supplementary Table S5).

#### 3.5.2. Expanded and contracted gene families

**Figure 8.**
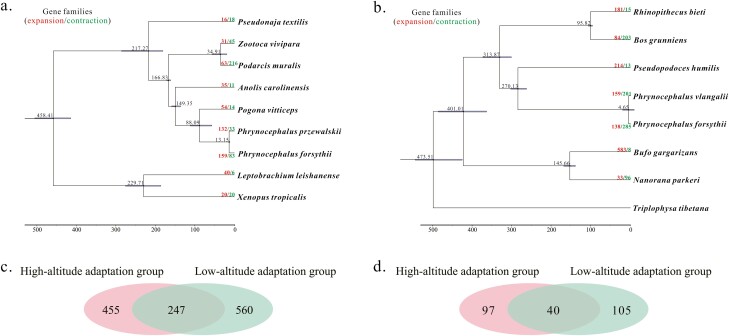
Comparison of gene families. Gene family expansion and contraction in each evolutionary branch of high-altitude adaptation group (A) and low-altitude adaptation group (B). The numbers of expanded and contracted gene families are shown at the nodes in the phylogenetic tree. The divergence time is given in millions of years. (C): Venn diagrams of genes from *P. forsythii* expansion gene family in the high-altitude adaptation group and low-altitude adaptation group. (D) Venn diagrams of genes from *P. forsythii* contraction gene family in the high-altitude adaptation group and low-altitude adaptation group.

In the high-altitude adaptation group, a total of 159 gene families were significantly expanded and 83 gene families contracted in the *P. forsythii* genome (Fig. 8A). After removing common genes with low-altitude adaptation group in expanded and contracted families, 455 genes from expanded gene families and 97 genes from contracted gene families were retained (Fig. 8C and D). These genes were executed for GO enrichment analysis, and were over-represented in some major molecular functional categories. In expanded gene families, genes were mainly enriched in DNA methylation or demethylation (DNA demethylation, DNA methylation or demethylation, oxidative DNA demethylation, oxidative single-stranded DNA demethylation, oxidative DNA demethylase), immune (antigen receptor-mediated signalling pathway, B-cell receptor signalling pathway, immune response-activating signal transduction, immune response-activating cell surface receptor signalling pathway, histone methyltransferase activity (H3-K4 specific) and immune response-activating cell surface receptor signalling pathway), energy metabolism (ATPase complex and ATP hydrolysis activity and ATP-dependent activity), and DNA repair (DNA dealkylation involved in DNA repair, DNA repair, cellular response to DNA damage stimulus, recombinational repair, and mismatch repair) (Supplementary Table S6). For contracted gene families, genes were mainly enriched in gene function related to signal transduction (molecular transducer activity, signalling receptor activity, transmembrane signalling receptor activity, and G protein-coupled receptor activity) (Supplementary Table S6 and Supplementary Fig. S1).

In the low-altitude adaptation group, 138 and 285 gene families were significantly expanded and contracted (Fig. 8B). After removing common genes with the high-altitude adaptation group, 560 and 105 genes were retained from expanded and contracted gene families, respectively (Fig. 8C and D). Of then, genes from expanded gene families were over-represented in energy metabolism (lipid transport, lipid localization, long-chain fatty acid transport, fatty acid transport) and immune (lysozyme activity and peptidoglycan muralytic activity), and genes from contracted gene families were mainly enriched in sensory perception (olfactory receptor activity, G protein-coupled receptor activity, transmembrane signalling receptor activity, signalling receptor activity, and molecular transducer activity) (Supplementary Table S7 and Supplementary Fig. S1).

#### 3.5.3. Positively selected genes

In total, 2,206 genes of 10336 single-copy genes were positively selected in the *P. forsythii* genome, these genes were significantly enriched in hydrolase activity (corrected *P* = 1.49e−02), cation binding (corrected *P* = 1.49e−02), metal ion binding (corrected *P* = 1.49e−02), peptidase activity (corrected *P* = 1.49e−02), nucleoside-triphosphatase regulator activity (corrected *P* = 4.35e−02), GTPase regulator activity (*corrected P* = 4.35e−02), and catalytic activity (corrected *P* = 4.72e−02)(Supplementary Table S8). Furthermore, we found that heat shock protein genes (*Hspa1a*) showed signals of positive selection.

## 4. Discussion

### 4.1. Karyotypes relationship of Chinese *Phrynocephalus*

Previous study has revealed that karyotype of Chinese *Phrynocephalus* species originated from the same mutual ancestor and divergence into two different numbers of types: 2*n* = 46 or 2*n* = 48.^1^ However, the karyotype evolutionary relationship between them has not been elucidated. In this study, we present a chromosome-level genome of *P. forsythii* which could provide some new insights into the chromosome relationships of Chinese *Phrynocephalus* species.

Twelve microchromosomes of *P. forsythii* (marked in the green box in Fig. 3C) exhibit strong interactions between chromosomes based on the Hi-C heat map. However, unlike the 12 microchromosomes mentioned above, there is a single microchromosome (chromosome 24, marked with a blue box in Fig. 3C) that has a strong interaction with a macrochromosome (chromosome 11, interaction was marked with red arrow in Fig. 3C). Hi-C is a powerful tool for detecting chromosomal rearrangements, which can be detected by interaction map between chromosomes located off the diagonal of the Hi-C heat map.^43^ Therefore, strong interaction between chromosome 11 and chromosome 24 of *P. forsythii* suggested that the two chromosomes may be fissioned from a single chromosome. To test our idea, we performed the genome synteny analysis between *P. forsythii* and *Phrynosoma platyrhinos.* The result showed that *P. forsythii* chromosome 11 and chromosome 24 exhibited genome synteny with one chromosome of *P. platyrhinos*, indicating that the two chromosomes of *P. forsythii* belong to one in genome of *P.platyrhinos* (Fig. 6B and 6C). Furthermore, genome alignment analysis between *P. forsythii* (2*n* = 48) and *P. przewalskii* (2*n* = 46) showed that although the number of chromosomes differs between the two species,^1^ almost all *P. przewalskii* scaffolds could align to 48 chromosomes of *P. forsythii,* indicating that the genomes of the two species have certain homology, otherwise the extra chromosome of *P. forsythii* could not be aligned by *P. przewalskii.* Therefore, we speculate that chromosomes 11 and 24 are a single chromosome in Chinese *Phrynocephalus* 2*n* = 46 species as well as species in other genera, and this chromosome breaks into two with the speciation of *P. forsythii*, which may also be the cause of the extra pair of chromosomes in the viviparous clade species.

Novel microchromosomes created by macrochromosome fission have also been observed in other squamate reptiles, although such fission is much less common than the fusion of macro- and microchromosomes.^44^ Furthermore, although the number of macro- and microchromosomes varies among squamate reptiles, sequence comparisons show that macro- and microchromosomes of these species are almost wholly conserved.^45^ In conclusion, our research on the karyotype relationship of Chinese *Phrynocephalus* provides evidence that microchromosomes could originate from macrochromosome fission.

### 4.2. High-altitude adaptation evolution

Compared with other species distributed at lower altitudes, several gene families in *P. forsythii* show expansion relative to species inhabiting lower altitudes, indicative of high-altitude adaptation in *P. forsythii*. First, the most major adaptation is hypoxia response. When compared with other species living at low altitudes, *P. forsythii* species-specific genes were enriched in many GO categories related to calcium-ion transport. The calcium ion released from the sarcoplasmic reticulum is a principal determinant of cardiac contractility.^46^ In hypoxic environments, *P. forsythii* can enhance myocardial contractility by calcium-ion transport, thereby improving blood oxygen transport. Furthermore, many GO categories related to DNA methylation or demethylation have been found in *P. forsythii* expanded gene families. DNA methylation and demethylation enable animals to produce hypoxic responses and adaptation by regulating the transcription of hypoxia-response genes.^47^ These results indicate that hypoxia is one of the major challenges for *P. forsythii* to adapt to high-altitude environments. Second, *P. forsythii* has increased energy metabolism, probably in response to hypoxic and low environment temperatures condition. Hypoxia reduces the oxygen supply to the cells and severely limits aerobic metabolism, thereby exacerbating the energy metabolism supply of animals that are native to high-altitude environments.^48^ Furthermore, ectothermic animals whose body temperatures are highly dependent upon the environment face a more serious challenge at high altitudes, as their access to energy is further limited in these environments.^49^ For these, gene involved in aerobic metabolism are observed in expanded gene families in the *P. forsythii* genome. Third, *P. forsythii* has responded to UV radiation. Increased exposure to UV radiation in high-elevation environments can damage DNA molecules,^50^ so DNA damage response and repair pathways may show functional adaptations for high-altitude animals. Accordingly, we have found some *P. forsythii* genes from expanded gene families were enriched in GO categories related to DNA repair. Besides, immunity may also be affected by exposure to ultraviolet (UV) radiation. UV exposure in laboratory affects antigen presentation, inflammatory response, and cytokine Production.^51,52^ We identified genes that were significantly enriched in immunity categories in expanded gene families of *P. forsythii*, which indicated that high-altitude environment also weakens the immune system of *P. forsythii.* In summary, *P. forsythii* may have adapted to high-altitude environments by enhancing oxygen transport, energy, and immunological aspects. These may also be the genetic basis for the adaptation of other species in Chinese *Phrynocephalus* highland viviparous clade to the Tibetan plateau environment.

### 4.3. Low-altitude adaptation evolution

After originating at high altitudes, *P. forsythii* has subsequently spread and colonized the northern Tarim Basin,^10^ an area with high ambient temperatures, and the high ambient temperature is thought another form of environmental stress for *P. forsythii*. Our result indicated that the genome of *P.forsythii* may have produced adaptive characteristics for the high ambient temperature environment. Firstly, compared with other species living at high altitudes, some *P. forsythii* genes from expanded gene families are enriched in lysozyme activity, which functions about antibacterial defence in animals are widely recognized.^53^ This indicated that the high ambient temperature has reduced the survival condition of low-altitude populations of *P. forsythii* and that bacteria are at higher densities at higher ambient temperatures, these conditions may lead to *P. forsythii* being more susceptible to bacterial invasion. This result is also consistent with our previous research, in which a larger number of pathogenic bacteria were found in the low-altitude population of *P. forsythii*.^54^ Furthermore, many species-specific genes of *P. forsythii* compared with other species living at high altitudes are enriched in many GO categories related to immune, which also indicated *P. forsythii* may respond to the infestation of these bacteria. Second, *P. forsythii* has also increased energy metabolism in high ambient temperature environments, and some enriched GO categories in *P. forsythii* expanded gene families are related to metabolism. As ectothermic, *P. forsythii* body temperature is highly dependent on the environment. In localities where ambient temperatures exceed the critical thermal maximum, ectotherms will retreat to thermal refugia on a daily basis, and therefore reducing foraging time and, hence, energy intake is therefore limited.^7^ Finally, positive selection gene *Hspa1a* has been found in *P. forsythii* genome. These genes belong to heat shock proteins (Hsp), when organisms are exposed to heat, cold, or some other stresses, they can synthesize Hsps which participate in unfolding and relocalization of proteins damaged by the stresses.^55^ These results indicated that *P. forsythii* has been stressed by the high-temperature environment and responded accordingly.

## 5. Conclusions

Based on Illumina short reads, Oxford Nanopore long reads, and Hi-C data, we generated a chromosome-level reference genome with a size of 1.82 Gb for *P. forsythii*. This chromosome-level genome indicated that two chromosomes of *P. forsythii* were originated from one ancestral chromosome of species with 46 chromosomes. Comparative genomic analysis revealed that *P. forsythii* genome has changed for coping with the life in the high-altitude extreme environment since it originated in the high altitude. In subsequent evolutionary history, *P. forsythii* has spread to a high ambient temperature environment, and the *P. forsythii* genome has produced some relevant adaptive characteristics. The genomic resources of *P. forsythii* and those candidate genes related to extreme environment adaptation provide more resources and insights about understanding the evolutionary development and ecological genomics of reptiles.

## Supplementary Material

dsad003_suppl_Supplementary_Figure_S1

dsad003_suppl_Supplementary_Table_S1

dsad003_suppl_Supplementary_Table_S2

dsad003_suppl_Supplementary_Table_S3

dsad003_suppl_Supplementary_Table_S4

dsad003_suppl_Supplementary_Table_S5

dsad003_suppl_Supplementary_Table_S6

dsad003_suppl_Supplementary_Table_S7

dsad003_suppl_Supplementary_Table_S8

## Data Availability

Raw data from *de novo* sequencing are available through the NCBI Sequence Read Archive (SRA) under projects PRJNA699736. Genome sequences and annotation files are deposited in the NCBI database under accession number JAPFRF000000000.

## References

[1] Zeng, X., Wang, Y., Liu, Z., et al. 1997, Karyotypes of nine species in the genus *Phrynocephalus*, with discussion of karyotypic evolution of Chinese *Phrynocephalus*, Acta Zool. Sin., 43, 399–410.

[2] Qiu, Q., Zhang, G., Ma, T., et al. 2012, The yak genome and adaptation to life at high altitude, Nat. Genet., 44, 946–9. 22751099 10.1038/ng.2343

[3] Qu, Y., Zhao, H., Han, N., et al. 2013, Ground tit genome reveals avian adaptation to living at high altitudes in the Tibetan plateau, Nat. Commun., 4, 2071. 23817352 10.1038/ncomms3071

[4] Li, J., Gao, Y., Xie, L., et al. 2018, Comparative genomic investigation of high-elevation adaptation in ectothermic snakes, Proc. Natl. Acad. Sci. U. S. A., 115, 8406–11. 30065117 10.1073/pnas.1805348115 PMC6099860

[5] Deutsch, C.A., Tewksbury J.J., Huey, R.B, et al. 2008, Impacts of climate warming on terrestrial ectotherms across latitude, Proc. Natl. Acad. Sci. U. S. A., 105, 6668–72. 18458348 10.1073/pnas.0709472105 PMC2373333

[6] Dillon, M.E., Wang, G., and Huey, R.B. 2010, Global metabolic impacts of recent climate warming, Nature, 467, 704–6. 20930843 10.1038/nature09407

[7] Sinervo, B., Miles, D.B., Wu, Y., Méndez-DE LA Cruz, F.R., Kirchhof, S., and Qi, Y. 2018, Climate change, thermal niches, extinction risk and maternal-effect rescue of toad-headed lizards, *Phrynocephalus*, in thermal extremes of the Arabian Peninsula to the Qinghai-Tibetan Plateau, Integr. Zool., 13, 450–70. 29436768 10.1111/1749-4877.12315

[8] Jin, Y., and Brown, R.P. 2013, Species history and divergence times of viviparous and oviparous Chinese toad-headed sand lizards (*Phrynocephalus*) on the Qinghai-Tibetan Plateau, Mol. Phylogenet. Evol., 68, 259–68. 23567019 10.1016/j.ympev.2013.03.022

[9] Jin, Y., Wo, Y.B., Tong, H.J., Song, S., Zhangm, L.X., and Brown, R.P. 2018, Evolutionary analysis of mitochondrially encoded proteins of toad-headed lizards, *Phrynocephalus*, along an altitudinal gradient, BMC Genomics, 19, 185. 29510674 10.1186/s12864-018-4569-1 PMC5840783

[10] Qi, Y., Zhao, W., Li, Y., and Zhao, Y. 2020, Environmental and geological changes in the Tarim Basin promoted the phylogeographic formation of *Phrynocephalus forsythii* (Squamata: Agamidae), Gene, 768, 145264. 33129850 10.1016/j.gene.2020.145264

[11] Qi, Y., Zhao, W., Huang, Y., et al. 2019, Correlation between Climatic Factors and Genetic Diversity of *Phrynocephalus forsythii*, Asian Herpetol. Res., 10, 270–5.

[12] Gao, W., Sun, Y., Zhou, W., et al. 2018, Genomic and transcriptomic investigations of the evolutionary transition from oviparity to viviparity, Proc. Natl. Acad. Sci. U. S. A., 116, 3646–55. 10.1073/pnas.1816086116 PMC6397529 30808754

[13] Marcais, G. and Kingsford, C. 2011, A fast, lock-free approach for efficient parallel counting of occurrences of k-mers, Bioinformatics, 27, 764746–770. 10.1093/bioinformatics/btr011 PMC3051319 21217122

[14] Luo, R., Liu, B., Xie, Y., et al. 2012, SOAPdenovo2: an empirically improved memory-efficient short-read *de novo* assembler, GigaScience, 1, 1–18. 23587118 10.1186/2047-217X-1-18 PMC3626529

[15] Ruan, J. and Li, H. 2020, Fast and accurate long-read assembly with wtdbg2, Nat. Methods, 17, 155–8. 31819265 10.1038/s41592-019-0669-3 PMC7004874

[16] Walker, B.J., Abeel, T., Shea, T., et al. 2014, Pilon: an integrated tool for comprehensive microbial variant detection and genome assembly improvement, PLoS One, 9, e112963. 25409509 10.1371/journal.pone.0112963 PMC4237348

[17] Zhang, X., Zhang, S., Zhao, Q., Ming, R., and Tang, H. 2019, Assembly of allele-aware, chromosomal-scale autopolyploid genomes based on Hi-C data, Nat. Plants, 5, 833–45. 31383970 10.1038/s41477-019-0487-8

[18] Simao, F.A., Waterhouse, R.M., Ioannidis, P., Kriventseva, E.V., and Zdobnov, E.M. 2015, BUSCO: assessing genome assembly and annotation completeness with single-copy orthologs, Bioinformatics, 31, 3210–2. 26059717 10.1093/bioinformatics/btv351

[19] Parra, G., Bradnam, K., and Korf, I. 2007, CEGMA: a pipeline to accurately annotate core genes in eukaryotic genomes, Bioinformatics, 23, 1061–7. 17332020 10.1093/bioinformatics/btm071

[20] Li, H. and Durbin, R. 2009, Fast and accurate short read alignment with Burrows-Wheeler transform, Bioinformatics, 25, 1754–60. 19451168 10.1093/bioinformatics/btp324 PMC2705234

[21] Tarailo-Graovac, M. and Chen, N. 2009, Using RepeatMasker to identify repetitive elements in genomic sequences, Curr. Protoc. Bioinformatics, Chapter 4, Unit 4.10. 10.1002/0471250953.bi0410s25 19274634

[22] Majoros, W.H., Pertea, M., and Salzberg, S.L. 2004, TigrScan and GlimmerHMM: two open source *ab initio* eukaryotic gene-finders, Bioinformatics, 20, 2878–9. 15145805 10.1093/bioinformatics/bth315

[23] Korf, I. 2004, Gene finding in novel genomes, BMC Bioinf., 5, 59. 10.1186/1471-2105-5-59 PMC421630 15144565

[24] Gertz, E.M., Yu, Y.K., Agarwala, R., Schäffer, A.A., and Altschul, S.F. 2006, Composition-based statistics and translated nucleotide searches: improving the TBLASTN module of BLAST, BMC Biol., 4, 41. 17156431 10.1186/1741-7007-4-41 PMC1779365

[25] Birney, E., Clamp, M., and Durbin, R. 2004, GeneWise and genomewise, Genome Res., 14, 988–95. 15123596 10.1101/gr.1865504 PMC479130

[26] Haas, B.J., Papanicolaou, A., Yassour, M., et al. 2013, *De novo* transcript sequence reconstruction from RNA-seq using the Trinity platform for reference generation and analysis, Nat. Protoc., 8, 1494–512. 23845962 10.1038/nprot.2013.084 PMC3875132

[27] Haas, B.J., Salzberg, S.L., Zhu, W., et al. 2008, Automated eukaryotic gene structure annotation using EVidenceModeler and the Program to Assemble Spliced Alignments, Genome Biol., 9, R7. 18190707 10.1186/gb-2008-9-1-r7 PMC2395244

[28] Zdobnov, E.M. and Apweiler, R. 2001, InterProScan - an integration platform for the signature-recognition methods in InterPro, Bioinformatics, 17, 847–8. 11590104 10.1093/bioinformatics/17.9.847

[29] Finn, R.D., Bateman, A., Clements, J., et al. 2014, Pfam: the protein families database, Nucl. Acids Res., 42, D222–230. 24288371 10.1093/nar/gkt1223 PMC3965110

[30] Lowe, T.M. and Eddy, S.R. 1997, tRNAscan-SE: a program for improved detection of transfer RNA genes in genomic sequence, Nucleic Acids Res., 25, 955–64. 9023104 10.1093/nar/25.5.955 PMC146525

[31] Griffiths-Jones, S., Bateman, A., Marshall, M., Khanna, A., and Eddy, S.R. 2003, Rfam: an RNA family database, Nucleic Acids Res., 31, 439–41. 12520045 10.1093/nar/gkg006 PMC165453

[32] Kurtz, S., Phillippy, A., Delcher, A.L., et al. 2004, Versatile and open software for comparing large genomes, Genome Biol., 5, R12. 14759262 10.1186/gb-2004-5-2-r12 PMC395750

[33] Koochekian, N., Ascanio, A., Farleigh, K., et al. 2022, A chromosome-level genome assembly and annotation of the desert horned lizard, *Phrynosoma platyrhinos*, provides insight into chromosomal rearrangements among reptiles, GigaScience, 11, 1–14. 10.1093/gigascience/giab098 PMC8848323 35134927

[34] Li, L., Stoeckert, C.J., and Roos, D.S. 2003, OrthoMCL: identification of ortholog groups for eukaryotic genomes, Genome Res., 13, 2178–89. 12952885 10.1101/gr.1224503 PMC403725

[35] Maere, S., Heymans, K., and Kuiper, M. 2005, BiNGO: a Cytoscape plugin to assess overrepresentation of gene ontology categories in biological networks, Bioinformatics, 21, 3448–9. 15972284 10.1093/bioinformatics/bti551

[36] Smoot, M.E., Ono, K., Ruscheinski, J., Wang, P.-L., and Ideker, T. 2011, Cytoscape 2.8: new features for data integration and network visualization, Bioinformatics, 27, 431–2. 21149340 10.1093/bioinformatics/btq675 PMC3031041

[37] Katoh, K. and Standley, D.M. 2001, MAFFT multiple sequence alignment software version 7: improvements in performance and usability, Mol. Biol. Evol., 30, 772–80. 10.1093/molbev/mst010 PMC3603318 23329690

[38] Talavera, G. and Castresana, J. 2007, Improvement of phylogenies after removing divergent and ambiguously aligned blocks from protein sequence alignments, Syst. Biol., 56, 564–77. 17654362 10.1080/10635150701472164

[39] Abascal, F., Zardoya, R., and Posada, D. 2005, ProtTest: selection of best-fit models of protein evolution, Bioinformatics, 2, 2104–5. 10.1093/bioinformatics/bti263 15647292

[40] Stamatakis, A. 2006, RAxML-VI-HPC: maximum likelihood-based phylogenetic analyses with thousands of taxa and mixed models, Bioinformatics, 22, 2688–90. 16928733 10.1093/bioinformatics/btl446

[41] Yang, Z. 2007, PAML 4: phylogenetic analysis by maximum likelihood, Mol. Biol. Evol., 24, 1586–91. 17483113 10.1093/molbev/msm088

[42] Suyama, M., Torrents, D., and Bork, P. 2006, PAL2NAL: robust conversion of protein sequence alignments into the corresponding codon alignments, Nucleic Acids Res., 34, W609–12. 16845082 10.1093/nar/gkl315 PMC1538804

[43] Harewood, L., Kishore, K., Eldridge, M.D., et al. 2017, Hi-C as a tool for precise detection and characterisation of chromosomal rearrangements and copy number variation in human tumours, Genome Biol., 18, 125. 28655341 10.1186/s13059-017-1253-8 PMC5488307

[44] Waters, P.D., Patel, H.R., Ruiz-Herrera, A., et al. 2021, Microchromosomes are building blocks of bird, reptile, and mammal chromosomes, Proc. Natl. Acad. Sci. U. S. A., 118, e2112494118. 34725164 10.1073/pnas.2112494118 PMC8609325

[45] O’Connor, R.E., Romanov, M.N., Kiazim, L.G., et al. 2018, Reconstruction of the diapsid ancestral genome permits chromosome evolution tracing in avian and non-avian dinosaurs, Nat. Commun., 9, 1883. 29784931 10.1038/s41467-018-04267-9 PMC5962605

[46] Gyorke, S., Gyorke, I., Lukyanenko, V., et al. 2002, Regulation of sarcoplasmic reticulum calcium release by luminal calcium in cardiac muscle, Front Biosci., 7, 1454–63. 10.2741/A852 12045014

[47] Watson, J.A., Watson, C.J., McCann, A., and Baugh, J. 2010, Epigenetics, the epicenter of the hypoxic response, Epigenetics, 5, 293–6. 20418669 10.4161/epi.5.4.11684

[48] Cheviron, Z.A. and Brumfield, R.T. 2012, Genomic insights into adaptation to high-altitude environments, Heredity, 108, 354–61. 21934702 10.1038/hdy.2011.85 PMC3313048

[49] Stanislaw, B., Mariusz, C., Ulf, B., et al. 2018, High oxidative stress despite low energy metabolism and vice versa: insights through temperature acclimation in an ectotherm, J. Therm. Biol., 78, 36–41. 30509659 10.1016/j.jtherbio.2018.08.003

[50] Svobodová, A.R., Galandáková, A., Sianská, J., et al. 2012, DNA damage after acute exposure of mice skin to physiological doses of UVB and UVA light, Arch. Dermatol. Res., 304, 407–12. 22271212 10.1007/s00403-012-1212-x

[51] Ullrich, S.E. and Schmitt, D.A. 2000, The role of cytokines in UV-induced systemic immune suppression, J. Dermatol. Sci., 1, S10–S12. 10.1016/s0923-1811(99)00073-0 10764984

[52] Cooper, K.D., Oberhelman, L., Hamilton, T.A., et al. 1992, UV exposure reduces immunization rates and promotes tolerance to epicutaneous antigens in humans: relationship to dose, CD1a-DR+ epidermal macrophage induction, and Langerhans cell depletion, Proc. Natl. Acad. Sci. U. S. A., 89, 8497–501. 1382291 10.1073/pnas.89.18.8497 PMC49947

[53] Callewaert, L. and Michiels, C.W. 2010, Lysozymes in the animal kingdom, J. Biosci., 35, 127–60. 20413917 10.1007/s12038-010-0015-5

[54] Qi, Y., Zhao, W., Zhao, Y., et al. 2020, The role of environmental stress in determining gut microbiome: case study of two sympatric toad-headed lizards, Asian Herpetol. Res., 11, 373–80.

[55] Goto, S.G. and Kimura, M.T. 1998, Heat- and cold-shock responses and temperature adaptations in subtropical and temperate species of *Drosophila*, J. Insect Physiol., 44, 1233–9. 12770323 10.1016/s0022-1910(98)00101-2

