# Supplementary material for: Chromosome-level genome assembly of Phrynocephalus forsythii using third-generation DNA sequencing and Hi-C analysis
Source: DNA Res. 2023 Mar 6;30(2):dsad003. doi: 10.1093/dnares/dsad003 (PMC10113879; doi:10.1093/dnares/dsad003)
Supplement: dsad003_suppl_Supplementary_Figure_S1 [file dsad003_suppl_supplementary_figure_s1.pdf]

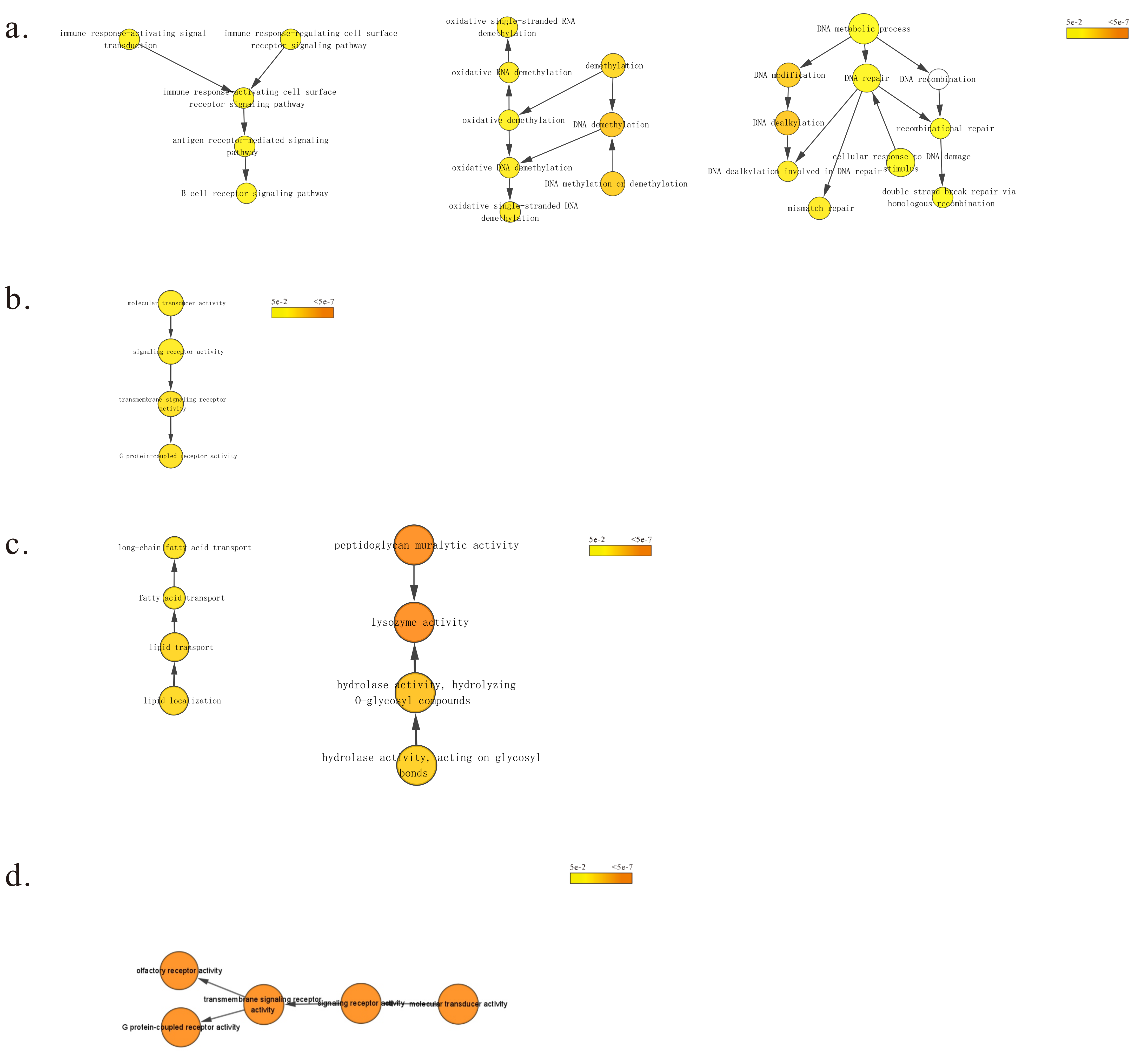

Figure S1: Overrepresented GO terms of *Phrynocephalus forsythii* genes from expanded or contracted gene families. Circle size is proportional to the number of genes in each category, whereas the color relates to the *P* value for the statistical significance of the enrichment. (a) Genes from expanded gene families in high-altitude adaptation group; (b) Genes from contracted gene families in high-altitude adaptation group; (c) Genes from expanded gene families in low-altitude adaptation group; (d) Genes from contracted gene families in low-altitude adaptation group.
